# Incidence rates and management of urinary tract infections among children in Dutch general practice: results from a nation-wide registration study

**DOI:** 10.1186/1471-2431-6-10

**Published:** 2006-04-04

**Authors:** Wing-Yee Kwok, Marjolein CE de Kwaadsteniet, Mirjam Harmsen, Lisette WA van Suijlekom-Smit, François G Schellevis, Johannes C van der Wouden

**Affiliations:** 1 Department of General Practice, Erasmus MC - University Medical Center Rotterdam, PO Box 1738, 3000 DR, Rotterdam The Netherlands; 2 Centre for Quality of Care Research (WOK), Radboud University Nijmegen Medical Centre, PO Box 9101, 6500 HB Nijmegen, The Netherlands; 3 Department of Paediatrics, Erasmus MC - University Medical Center/Sophia Children's Hospital, PO Box 1738, 3000 DR Rotterdam, The Netherlands; 4 Netherlands Institute for Health Services Research (NIVEL), PO Box 1568, 3500 BN Utrechtt the Netherlands

## Abstract

**Background:**

We aimed to investigate incidence rates of urinary tract infections in Dutch general practice and their association with gender, season and urbanisation level, and to analyse prescription and referral in case of urinary tract infections.

**Method:**

During one calendar year, 195 general practitioners in 104 practices in the Netherlands registered all their patient contacts. This study was performed by the Netherlands Institute for Health Services Research (NIVEL) in 2001. Of 82,053 children aged 0 to 18 years, the following variables were collected: number of episodes per patient, number of contacts per episode, month of the year in which the diagnosis of urinary tract infection was made, age, gender, urbanisation level, drug prescription and referral.

**Results:**

The overall incidence rate was 19 episodes per 1000 person years. The incidence rate in girls was 8 times as high as in boys. The incidence rate in smaller cities and rural areas was 2 times as high as in the three largest cities. Throughout the year, incidence rates varied with a decrease in summertime for children at the age of 0 to 12 years. Of the prescriptions, 66% were in accordance with current guidelines, but only 18% of the children who had an indication were actually referred.

**Conclusion:**

This study shows that incidence rates of urinary tract infections are not only related to gender and season, but also to urbanisation. General practitioners in the Netherlands frequently do not follow the clinical guidelines for urinary tract infections, especially with respect to referral.

## Background

Urinary tract infections in childhood are common and may be difficult to diagnose in young children because of non-specific symptoms. Symptoms such as fever, vomiting, screaming, anorexia and irritability may indicate a urinary tract infection, but they are also common in other childhood diseases [1,2].

The incidence of urinary tract infections during childhood is not only influenced by age, but also by gender. Before the age of 3 months, urinary tract infections are more common in boys; thereafter the incidence is considerably higher in girls [3,4]. In younger children, urinary tract infections are mainly caused by autoinfection with commensals from the intestinal tract [5], whereas urinary tract infections in adolescent girls are often related to sexual activity [6].

Reported incidence rates of childhood urinary tract infections vary. Table 1 shows incidence rates and study characteristics of eight studies that covered a substantial age range [2,7-13]. For boys the reported incidence rates range from 0.17 to 18 per 1000 person years and for girls from 0.4 to 66 per 1000 person years. This variation may be explained by differences in setting, health care system, age range, case definition, or study period. The range of occurrence rates found in the studies carried out in general practice is much smaller.

**Table 1:** Incidence rates of urinary tract infections in children reported in previous studies

**author**	**country, period**	**setting**	**age group**	**incidence in boys per 1000**	**incidence in girls per 1000**
Stansfield 1966^7^	UK 1957–1964	child referred to hospital for UTI	0–14	0.17	0.4
Winberg 1974^8^	Göteborg 1960–1966	hospital	0–11	11	30
Uhari 1988^10^	Finland 1978–1984	hospital admission for UTI	0–14	2	7.5
Pead 1994^11^	UK 1991–1992	culture requests positive	0–12	13.9	37
Mårild 1998^9^	Sweden 1979–1981	UTI diagnosed in hospital	0–6	18	66
Brooks 1977^12^	UK 1970–1974	UTI diagnosed in general practice	0–14	3.8	7.7
Dickinson 1979^13^	UK period unknown	UTI diagnosed in general practice	0–14	1.7	3.1
Van de Lisdonk 2000^2^	Netherlands 1971–1990	diagnosed in general practice	0–12	2.9	24.3

Urinary tract infections in children are an important cause of renal damage and chronic renal failure. Early diagnosis, treatment, investigation and follow-up of children with urinary tract infections are likely to reduce such long-term complications [14,15]. A guideline developed by the Dutch College of General Practitioners is available [16]. According to this guideline, nitrite testing should be performed, which, in case of a negative outcome, should be followed by a culture. Medication of first choice for children under the age of 12 years is a combination of amoxicillin and clavulanic acid or co-trimoxazole. For children older than 12 years, trimethoprim and nitrofurantoin are alternatives. The following patients should be referred for further investigation: all infants under the age of 1 year, all boys under the age of 12 years, girls aged 1 to 5 years with only one recurrence and girls aged 5 to 12 years with more than one recurrence.

The frequency of urinary tract infections in relation to age and gender, as well as diagnostic procedures and treatment were investigated in several studies [1,2,4,9,17-19]. However, little is known about seasonal variation and urbanisation. Seasonal variations in the incidence of human disease are commonplace, but in the case of urinary tract infections this aspect has rarely been addressed [7,9,20]. Therefore, our first aim is to describe incidence rates of urinary tract infections among children in Dutch general practice in 2001 by age, gender and in relation to season and urbanisation level. It is important to know if there is a relation between these factors and urinary tract infections, because this could provide general practitioners information about patients in which they should be more alert for urinary tract infections. In addition, we are interested if general practitioners follow the clinical guideline for urinary tract infections with respect to prescription and referral.

## Methods

### Design

We used data from the Second Dutch National Survey of General Practice, which was performed by the Netherlands Institute for Health Services Research (NIVEL) between April 2000 and January 2002 [21,22]. During the study period, 195 general practitioners in 104 practices throughout the Netherlands prospectively registered data about all their patient contacts during one calendar year. The participating general practitioners and practices were representative for all Dutch general practitioners and practices with regard to age, gender and location, including deprived areas.

In the Dutch health care system, general practitioners act as gatekeepers for secondary care. Every Dutch citizen is registered in general practice.

### Measurements

For socio-demographic characteristics (among which urbanisation) a short questionnaire was used and sent by mail to all patients registered in the participating practices (n = 385,461). Response on the questionnaire was 76%.

Characteristics, such as date of contact, type of contact, diagnosis (coded by the GP using the International Classification of Primary Care, ICPC) [23], drug prescription and referrals by the general practitioner, were registered for each contact by the general practitioner. The contact database was episode orientated, meaning that different consultations concerning the same health problem were clustered into one episode [21]. Only new episodes, starting during the registration period, were included in our analysis. We excluded eight practices, because of incomplete registration.

### Patients

Data were used from all children who were aged 0 to 18 years any time during the registration period. Of these, we selected all children with the diagnosis urinary tract infection (ICPC-code U71). This code excludes pyelonephritis, but does include recurring infections [19].

For all selected cases, the following variables were examined: number of episodes per patient, number of contacts per episode, month of the year in which the diagnosis was made, age, gender and urbanisation. Urbanisation was divided into the following four categories: < 30,000 inhabitants, 30,000–50,000 inhabitants, > 50,000 inhabitants and the 3 largest cities (Amsterdam, Rotterdam, The Hague).

### Ethical approval

The study was carried out according to Dutch legislation on privacy. The privacy regulation of the study was approved by the Dutch Data Protection Authority. According to Dutch legislation, obtaining informed consent is not obligatory for observational studies.

### Statistical analysis

Statistical analyses were performed using SPSS 11 and Stata 8 SE. We first computed the incidence rate of urinary tract infections in general practice. To determine the incidence rate, the denominator was calculated by summating the total time period children were followed during the study period (patientyears). The 95% confidence intervals of incidence rates were calculated assuming a Poisson distribution.

We analysed drug prescription and referral by general practitioners anywhere during the episode, comparing these to the recommendations in the guideline on urinary tract infections developed by the Dutch College of General Practitioners [16]. We used cut-off points for age with respect to prescription and referral, similar to the guideline for urinary tract infection of the Dutch College of General Practitioners.

## Results

The total patient population consisted of 385,461 persons, of which 82,053 were at the age of 0 to 18 years.

Of all episodes of children at the age of 0 to 18 years, 1.15% (1695 episodes) was diagnosed as a urinary tract infection (95% confidence interval [CI], 1.10–1.21).

Of all children with a urinary tract infection (n = 1460), the mean number of episodes per year was 1.2 per child, with a maximum of 5 episodes during the registration period of one year. For each episode of urinary tract infection, the mean number of consultations was 1.7, with a maximum of 13 consultations. The proportion of episodes with only one consultation was 66%.

**Figure 1:**
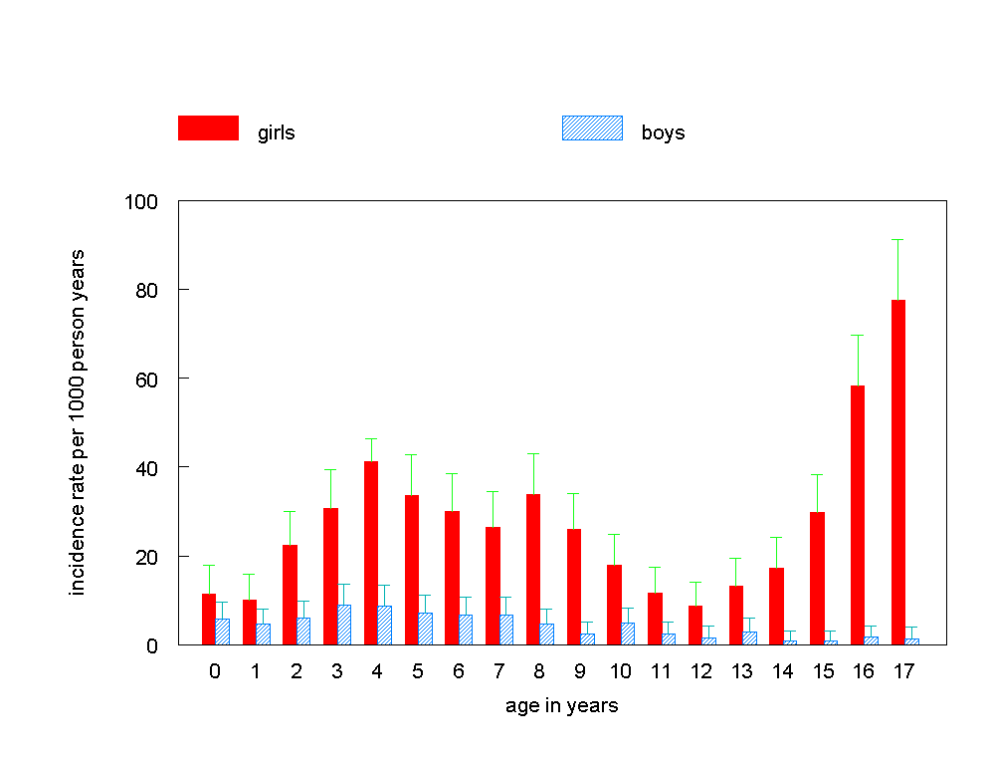
Incidence rates of urinary tract infections by age for boys and girls, 95% error bars.

We found an overall incidence rate of 19.0 episodes of urinary tract infections presented in general practice per 1000 person years for children under the age of 18 years (95% CI: 18.1–19.9). In other words; if 1000 children, aged 0 to 18 years, are followed for one year, their general practitioner will have made 19 times a diagnosis of a urinary tract infection. The incidence rate in girls was almost 8 times as high as in boys (respectively 34.4 episodes per 1000 person years vs. 4.4 episodes per 1000 person years, p < 0.001). Incidence rates of urinary tract infections for each year of age for both girls and boys are given in Figure 1. For girls the incidence gradually increased after the age of 12.

**Figure 2:**
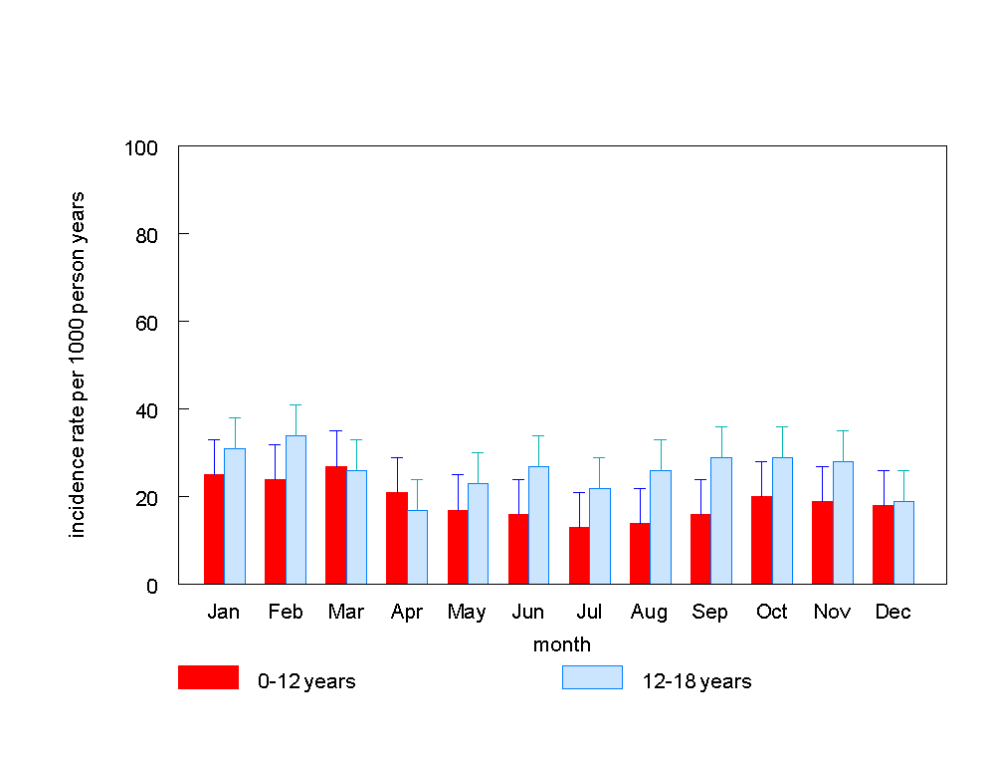
Incidence rates throughout the year by age group, 95% error bars.

**Figure 3:**
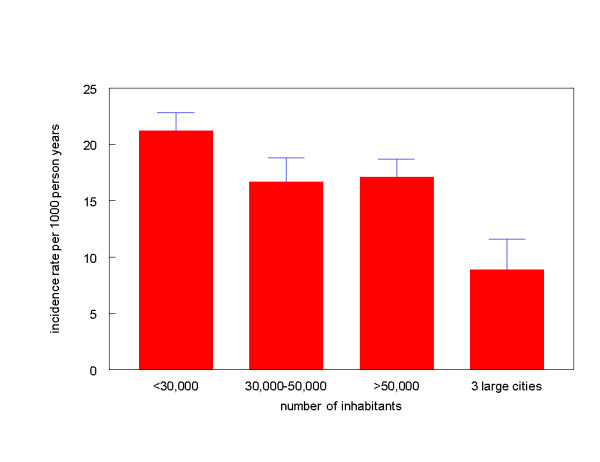
Incidence rates according to urbanisation, 95% error bars.

Furthermore, the incidence rates varied throughout the year. A decrease of incidence rates in summertime was mainly found in children at the age of 0 to 12 years, with a range from 27 episodes in March per 1000 person years to 13 episodes in July (Figure 2). The incidence rate in smaller cities and rural areas was 2.1 times as high as in the three largest cities, respectively 18.9 episodes per 1000 person years vs. 8.9 episodes per 1000 person years, p < 0.001 (Figure 3).

General practitioners prescribed medication for urinary tract infections 1776 times, to 1131 children (77% of all children in whom a urinary tract infection was diagnosed). When they chose to prescribe medication, 66% (1173 times) of the prescriptions were in accordance with the guideline of the Dutch College of General Practitioners. This percentage varied with age. The general practitioners deviated more frequently from the guideline for children under the age of 12 years than for children older than 12 years (Table 2). Children approaching the age of 12 years more often received medication for urinary tract infection advised in older children, such as trimethoprim and nitrofurantoin. Differences between boys and girls in the proportion of first choice medication were statistically significant for the first three age groups (p < 0.05), favouring girls for age groups 0–2 and 2–7, but favouring boys for age group 7–12.

**Table 2:** Number and proportion of prescriptions in accordance with the Dutch guideline for urinary tract infections, by age and sex group

age group (in years)	total number of prescriptions			prescriptions for medication of first choice number (%)		
	girls	boys	total	girls	boys	total
0 to 2	59	26	85	45 (76%)	12 (46%)	57 (67%)
2 to 7	485	83	568	261 (54%)	33 (40%)	294 (52%)
7 to 12	387	39	426	166 (43%)	26 (67%)	192 (45%)
12 to 18	669	28	697	605 (90%)	23 (82%)	628 (90%)
total	1600	176	1776	1077 (67%)	94 (53%)	1171 (66%)

In total 120 children were referred 134 times, of which 67% (90 times) to secondary care and 33% (44 times) to primary care. Of the children who should be referred according to the guideline (n= 225), only 18% (40 children) were actually referred. This percentage varied with age and gender (Table 3). Especially boys under the age of 12 were seldom referred (12%).

**Table 3:** Number of children to be referred accordancing to the guideline for urinary tract infections, and actual referrals, by age and sex group (n = 225)

category	number of children to be referred according to guideline	actual referrals *number (%)*
girls and boys, 0-year of age	42	13 (31%)
boys, aged 1 to 12 years	147	18 (12%)
girls, 1 recurrence, aged 1 to 5 years	27	6 (22%)
girls, > 1 recurrence, aged 5 to 12 years	9	3 (33%)
total	225	40 (18%)

The remaining 80 children had no indication for referral according to the guideline.

## Discussion

In this study, we found an overall incidence rate of 19 episodes of urinary tract infections presented by children in general practice per 1000 person years. This is strikingly similar to the incidence rate of 18.6 episodes per 1000 person years in 1987, found in the first National Survey, the design of which was very similar [22]. It appears that the incidence rate hardly changed over the past 15 years. Our results may be considered representative for all children treated in primary care in the Netherlands.

We found a significantly higher incidence rate for girls (34.4 episodes per 1000 person years) than for boys (4.4 episodes per 1000 person years). Earlier studies also showed that urinary tract infections are more common in girls, but the male/female ratio of incidence rates varied. Comparing our results to those of earlier studies in other countries (Table 1), shows that our findings for boys are comparable to the other studies in general practice (range 1.7–3.8), but for girls our results are much higher (range of other studies in general practice (3.1–24.3). Especially the UK studies performed in the seventies [12,13] show very low figures. A possible explanation of the higher incidence we found in girls than the Dutch study by Van de Lisdonk [2] is the greater age range in our study.

Our results showed a variation of the incidence rate throughout the year, with a decrease in summertime. This decrease was mainly found in children below the age of 12 years. Elo et al. and Stansfeld also found a decrease of incidence rates during the summer [3,7]. A possible explanation, suggested by Stansfeld, is that upper respiratory tract infections may precede urinary tract infections. Since cough and colds are more common in the winter months, the same would apply to urinary tract infections [7].

On the other hand, Jakobsson found a higher diagnostic rate during the summer months [20], and a Korean study found pyelonephritis incidence peaking in summer[24]. Mårild found no seasonal variation at all [9]. Differences in age groups and composition of study populations may contribute to these conflicting results. For example, the study of Jakobsson only collected information from children under the age of two years [20], whereas the children in the study of Mårild were younger than six years [9]. The age groups of children in the studies of Elo et al.[3] and Stansfeld [7] were more similar to our age group and the Korean study included patients of all ages [24]. Moreover, none of these studies were carried out in general practice, but obtained their information from pediatric centers or from health insurance claims data.

The incidence rate in smaller cities and rural areas is significantly higher than in the three largest cities. Although less clear, a similar trend was found in the first National Survey. An explanation could be that children with urinary tract infections in the three largest cities are more inclined to visit the hospital (emergency department) instead of the general practitioner. General practitioners in the three largest cities will miss these episodes during their registration period, resulting in a lower incidence rate. It is not likely that the discrepancy could be fully attributed to this explanation, therefore the clinical and epidemiological significance of urbanisation remains to be determined. A study carried out in Northern Norway (all ages) did not show a difference [25].

When general practitioners chose to prescribe medication for urinary tract infections, 66% of the prescriptions were in accordance with the guideline of the Dutch College of General Practitioners. This percentage varied with age. It is possible that general practitioners are less familiar with prescription of medication for urinary tract infections in younger children.

Our analyses showed, that only 18% of the referrals corresponded with this guideline. This could be an underestimation, because some of the patients may have had episodes of urinary tract infections before the registration period started. It appears that general practitioners are reluctant to refer children with urinary tract infections. It is also possible that they do not know the guideline or that they do not agree with it. Further investigations into this referral behaviour are necessary. Dependent on the findings, consequences for education of general practitioners may be considered.

## Conclusion

In conclusion, this study shows that incidence rates of urinary tract infections are not only related with gender and season, but also with urbanisation. Furthermore, general practitioners in the Netherlands do not always follow the guideline for urinary tract infections developed by the Dutch College of General Practitioners concerning prescription and referral. Especially the referral behavior of general practitioners differs considerably from the guideline.

## Competing interests

The author(s) declare that they have no competing interests.

## Authors' contributions

WYK and MCEdK wrote the protocol, carried out the statistical analyses and drafted the paper. FGS was primary investigator of the Second National Survey, JCvdW coordinated data retrieval and supervised the analyses and the preparation of the manuscript. All authors commented on draft versions of the paper and approved the final manuscript.

## Pre-publication history

The pre-publication history for this paper can be accessed here:


